# POI recommendation with queuing time and user interest awareness

**DOI:** 10.1007/s10618-022-00865-w

**Published:** 2022-10-03

**Authors:** Sajal Halder, Kwan Hui Lim, Jeffrey Chan, Xiuzhen Zhang

**Affiliations:** 1 School of Computing Technologies, RMIT University, Melbourne, Australia; 2 Singapore University of Technology and Design, Singapore, Singapore

**Keywords:** Points of Interest (POI), POI recommendation, Transformer, Multi-tasking, Multi-head attention, Queuing time, User interest

## Abstract

Point-of-interest (POI) recommendation is a challenging problem due to different contextual information and a wide variety of human mobility patterns. Prior studies focus on recommendation that considers user travel spatiotemporal and sequential patterns behaviours. These studies do not pay attention to user personal interests, which is a significant factor for POI recommendation. Besides user interests, queuing time also plays a significant role in affecting user mobility behaviour, e.g., having to queue a long time to enter a POI might reduce visitor’s enjoyment. Recently, attention-based recurrent neural networks-based approaches show promising performance in the next POI recommendation task. However, they are limited to single head attention, which can have difficulty in finding the appropriate user mobility behaviours considering complex relationships among POI spatial distances, POI check-in time, user interests and POI queuing times. In this research work, we are the first to consider queuing time and user interest awareness factors for next POI recommendation. We demonstrate how it is non-trivial to recommend a next POI and simultaneously predict its queuing time. To solve this problem, we propose a multi-task, multi-head attention transformer model called TLR-M_UI. The model recommends the next POIs to the target users and predicts queuing time to access the POIs simultaneously by considering user mobility behaviours. The proposed model utilises POIs description-based user personal interest that can also solve the new categorical POI cold start problem. Extensive experiments on six real-world datasets show that the proposed models outperform the state-of-the-art baseline approaches in terms of precision, recall, and F1-score evaluation metrics. The model also predicts and minimizes the queuing time. For the reproducibility of the proposed model, we have publicly shared our implementation code at GitHub (https://github.com/sajalhalder/TLR-M_UI).

## Introduction

POI recommendation problems have attracted researchers’ interest due to their economic and academic significance. Travel and tourism are popular leisure activities, which is also a trillion-dollar industry across the world (Statista 2018). The tourism industry remains an important source of income and employment in many countries, both formal and informal sectors (Malik et al. 2010). For example, Hwang et al. (Hwang and Lee 2019b) claimed that Korean development and economic growth are rapidly increasing due to changes in the elderly tourism system. This increase shows that visitors feel inner satisfaction, which positively affects their future tour plan (Hwang and Lee 2019a). Similarly, developing countries can boost their sustainable growth and development by engendering a considerable amount of foreign exchange from tourism. These extensive tourism-related services (i.e., hotels and restaurants reservations and travel mode fixations) depend on user intention and budget. Thus, it achieves researcher attention to make better user personalised services and contribute to global economic growth.

Most previous POI recommendation models regard user identity as an invariant feature. However, in real-world POI recommendations, user preference may change based on spatiotemporal features, queuing features and user interests. To improve the travel and tourism experience, appropriate POI recommendation based on tourist personalised interest has attracted much attention from researchers in recent years (Chang et al. 2018b; Huang et al. 2019; Yin et al. 2017; Ding and Chen 2018; Rahmani et al. 2020; Baral and Li 2018; Anagnostopoulos et al. 2017). These POI recommendations can be challenging because visitors^1^ often have multiple criteria and different preferences when choosing a POI to visit next. For example, some visitors may prefer the nearest available POI that they are mildly interested in, while others might prefer one that they are very interested in despite traveling a longer distance. Some visitors are interested in visiting their preferred POIs. Others may have dynamic preferences where their previous visits may not reflect their most recent interest preferences. Most of the deep learning techniques cannot handle multiple conflicts of immediate and long-distance priorities as well as recent and past visit influence simultaneously. LSTM or RNN based approaches focus on recent visits and closest preferences based on spatiotemporal dependencies. User interests do not always depend on closest preferences. Thus, learning user preference based on spatiotemporal dependencies can not capture user intentions appropriately. Besides, another factor that affects visitor’s satisfaction is the duration of queuing time.

**Fig. 1:**
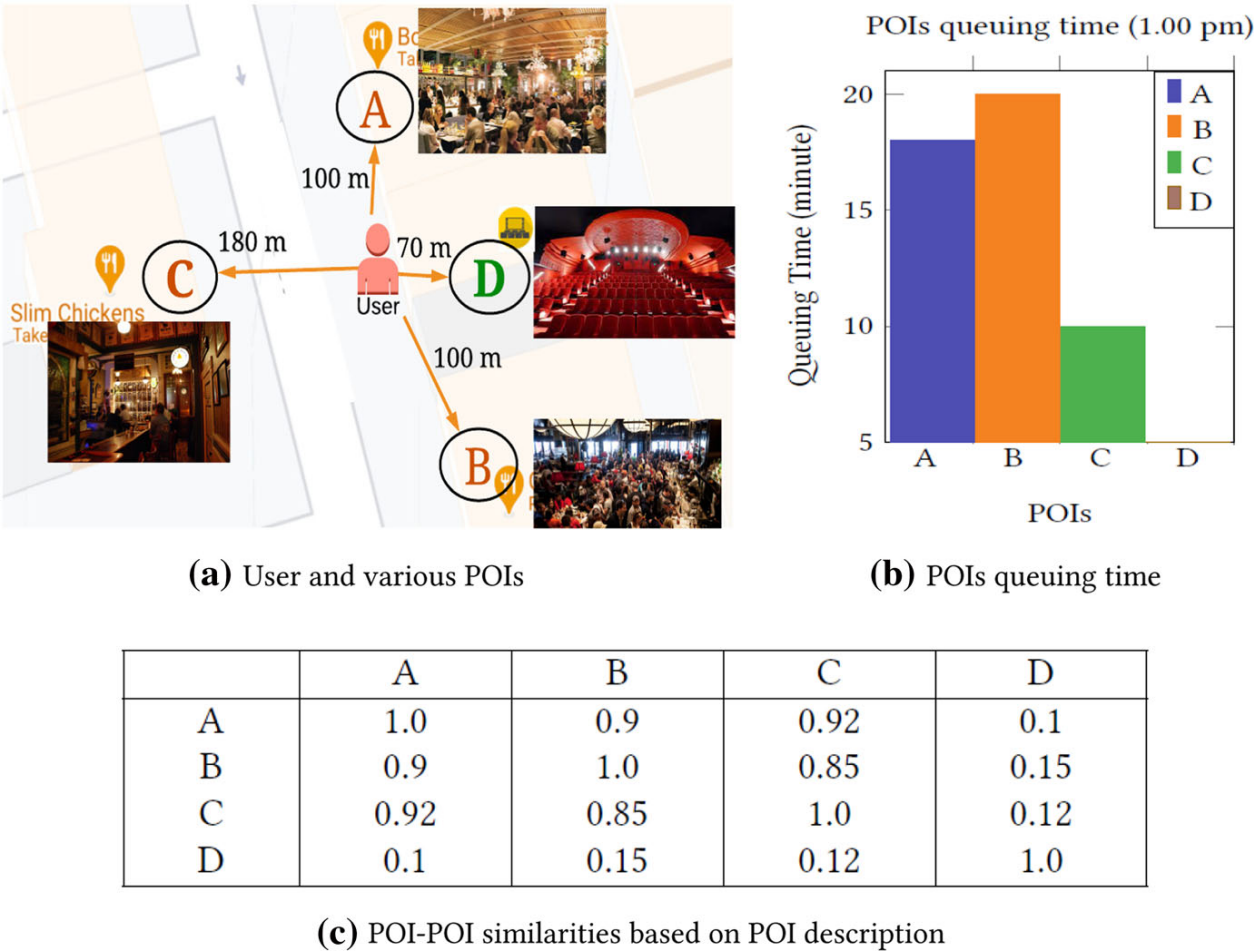
Influence of POI description and queuing time along with spatiotemporal features in POI recommendation

Figure 1 depicts an example showing the various POI similarities and significance of queuing time. Assuming that the current time is 1.00 pm and a visitor wants to go to a restaurant for lunch. If the next POI recommendation model does not consider POI description/category, it may recommend POI *D* because POI *D* is closer. Besides, if the model only considers POI description/category and does not consider queuing time of these POIs, it may recommend nearby restaurant *A* or *B* according to the distance. However, these two restaurants are crowded places, and users have to wait a long time to have their lunch which is generally undesirable. Besides this, users might not be interested in going far for lunch on weekdays due to office work, but they might be interested in going far on weekends. Thus, a queuing time-aware next POI recommendation model that takes into consideration POIs description, queuing information, spatiotemporal dependencies and personalised interest is more likely to recommend restaurant *C* to the user as the next move.

These kinds of queuing time-related activities are also prevalent and important in many other real-life applications, e.g., theme parks and popular tourist attractions, restaurants, concerts, and festivals. Besides, with the COVID-19 pandemic (COVID-19 2019), there is a need to maintain a physical distance, and queuing takes on a health dimension, making the queuing influence even more significant. Moreover, user interest based on POIs similarity plays significant roles in POI recommendation. Existing models (Lim et al. 2017; Halder et al. 2022) used POI categorical similarity to measure user interests where values are 1 or 0 (1 means two POIs are similar categories and 0 means two POIs are different categories). These categorical similarity-based user interests may not measure real-valued similarity and can not distinguish similarity levels within the same category. Figure 1c shows the similarities matrix among the four POIs where three POIs *A*, *B* and *C* are restaurants and POI *D* is cinema hall.

The categorical POI similarity also faces a cold start problem when POIs of a new category are added into the network. Although two POI categories are different, similar types of keywords may describe the POIs. Thus, instead of categorical similarities, description-based POIs similarities are able to solve the cold start problem. Thus, visited POI description-based similarity sequence may be practical to predict user movement behaviour patterns.

These challenges inspired us to build a model that can capture POIs description similarities, complex spatiotemporal dependencies, queuing time influence and user interests for making an accurate next POI recommendation. The problems of POI recommendation and queue time prediction are inter-dependent. Thus, a single model that jointly recommends top-k POIs and predicts queuing time is necessary.

Existing studies on POI recommendation have considered spatiotemporal preferences (Liu et al. 2016) but did not consider user preference. In another group of prior research, user identification is considered and attention-based spatiotemporal influence based ATST-LSTM (Huang et al. 2019) and self-attentive network SANST (Guo and Qi 2020) have been proposed. These works are appropriate for the next POI recommendation, but they cannot support multi-tasking (recommend POIs and predict queuing time) simultaneously. Recently, attention-based transformer shows significant improvement to capture all dependencies at once using non-recurrent encoder-decoder model in volatility prediction (Yang et al. 2020; Wu et al. 2020) and natural language processing (Devlin et al. 2018). Transformer allows multi-tasking that relies entirely on the attention mechanism to compute its input and output dependencies. In our early work TLR-M (Halder et al. 2021), we proposed multi-attention layer-based transformer network leveraging complex spatiotemporal dependencies whereas user personal interest based on temporal influence is ignored. To address this issue, we use POI description similarity based personalised interest to recommend POIs and predict queuing time simultaneously. The main difference between our proposed model and earlier transformer based multitasking model TLR-M (Halder et al. 2021) is that our model incorporates user interests from one POI to another POI based on POIs descriptions which can solve POI cold start problem whereas existing TLR-M (Halder et al. 2021) does not consider user interest. To sum up, in this paper we aim to answer the following research questions.

- Are user interests important for recommending top-k POIs?
- Are user interests important for recommending top-k POIs and predicting queuing times simultaneously?
- How does POI description based user interests perform compared to the POI category based user interests?

This work is an extension of our previous conference paper (Halder et al. 2021). Here we extend POI description based personalised user interest impacts on queuing time prediction and next POI recommendation simultaneously. In this paper, we consider user visiting POI similarity sequence for capturing use mobility behaviours appropriately. We propose two new models called TLR_UI and TLR-M_UI. Doc2vec (Le and Mikolov 2014) model has been used to measure the similarities between visited POIs sequences instead of only categorical similarities. To evaluate the performance of our proposed model, we compared our models to various state-of-the-art models on six datasets and discuss our main findings. The main contributions of this paper can be summarized as follows:

- This work discusses the significance of POI description-based user interests and queuing time aware next POI recommendation model. More specifically, the model captures user interest behavior along with spatiotemporal and queuing time influences.
- We capture user interest from visited POIs description using POI description similarity measurement technique and applied to transformer network to enhance recommendation efficiency that can solve the POIs cold start problem.
- Experiment results using six real-life datasets show our proposed transformer model outperforms the state-of-the-art next personalised POI recommendation based on precision, recall, F1-score and can predict queuing time effectively.

The remaining parts of this study are organized as follows. The related works are described in Sect. 2. Sect. 3 introduces some preliminaries and problem statements. After that, we propose TLR-M_UI and TLR_UI models incorporating the user interest, queuing influence and spatiotemporal dependencies in Sect. 4.1 and Sect. 4.2, respectively. The experiment analyses with the state-of-the-art baselines are illustrated in Sect. 5. Finally, we conclude our proposed model with future work direction in Sect. 6.

## Related work

This research is related to research on top-k POI recommendation, queuing time prediction, transformer-based learning, and features embedding. In this section, we briefly describe state-of-the-art research related to our work in each of these areas. Then, we highlight the significant differences between our proposed models and the existing baselines.

### POI recommendation

POI recommendation accuracy depends on multiple factors. The previous study LORE (Zhang et al. 2014) incorporates geographical influence and social influence into a unified recommendation framework in the check-in dataset. Simultaneously, a convolutional LSTM (Xingjian et al. 2015) network has been proposed to solve temporal and spatial dependencies where user interest was ignored. Chang et al. (Chang et al. 2018a) proposed a deep neural POI imputation model called DeepPIM, which utilises textual, visual, user, and temporal features without complex pre-processing or feature engineering in item recommendation. Zheng et al. (Zheng et al. 2018) introduced a deep reinforcement learning framework to do online personalised news recommendations using state feature and action feature representation. These items and news recommendations are not appropriate for POI recommendations because spatial distance has a significant influence in POI recommendations. Chang et al. (Chang et al. 2018b) proposed a context-aware hierarchical POI embedding model CAPE for POI recommendation using the user check-in sequence and text content of POIs whereas personalised interest was not considered. Zhou et al. (Zhou et al. 2019b) introduced a more generic framework for POI recommendation that is sufficiently flexible to incorporate different contextual information, but the model does not support multitasking. A time-aware successive POI recommendation method STELLAR (Zhao et al. 2016) has been proposed to show the effects of three-slice time interval successive check-ins. Zhang et al. (Zhang and Chow 2015) proposed a probabilistic framework that concerns not only time slots of a day but also the day of weekdays and weekends. These models are generic and can not distinguish personalised interest. Debnath et al. (Debnath et al. 2018) presented a time-aware and preference-aware routes recommendation system. A temporal personalised model (TPM) (Wang et al. 2018) has been proposed to recommend spatial items introducing a new latent variable topic-region by using sequential influence, cyclic patterns, and personal interest. In these research, queuing time influence is ignored.

Some studies (Yang et al. 2017; Wang et al. 2017) have employed convolutional neural networks and multi-layer preceptors to POI recommendation. These models used POI images, which can not differentiate near and far POIs. Huang et al. (Huang et al. 2019) proposed an attention-based spatiotemporal long and short-term memory (ATST-LSTM) network for the next POI recommendation, in which user interest was ignored. They used user identity as user vectors that could not capture personalised interest appropriately. Zhou et al. (Zhou et al. 2019a) proposed a generative discriminator-based POI recommendation model that maximizes the learned probabilities distributions and optimizes the differences between recommended POIs and true check-ins. However, all of these studies are single task learning models which recommend only next POIs to the user based on different features. Therefore, we need one model that can recommend top-k POIs and predict queuing time simultaneously incorporating spatio-temporal features, queuing time and user interests.

### Queuing time prediction

Due to the COVID-19 pandemic (COVID-19 2019), queuing time has been highly significant even though researchers ignored queuing time influence in POI recommendation. Considering queuing time, Lim et al. (Lim et al. 2017) proposed PresQ algorithm whose objective is maximizing the POI popularity, user interest and minimizing the queuing time. Halder et al. (Halder et al. 2022) proposed an efficient EffiTourRec algorithm using personalised POI selection and pruning strategies to improve the performance of itinerary recommendation with queuing time awareness and visiting time influence. These queuing time-based approaches are applied in the whole itinerary recommendation. Therefore, none of the existing methods predicts queuing time considering user interests in the next POI recommendation. Thus, we introduce the queuing time and user interest aware prediction model that can recommend top-k POI and predict queuing time simultaneously.

### Transformer and multi-task learning

The transformer network-based model improves accuracy across a variety of NLP tasks (Devlin et al. 2018). The model can capture all word dependencies in a sentence to predict the next word. Recently, some research works in transformer-based model (Yang et al. 2020; Wu et al. 2020) show significant improvements in volatility prediction and event forecasting using multi-head attention technique. It has been shown that the transformer model is faster than the recurrent and convolutional layers-based models and improved performance using the multi-head self-attention technique (Vaswani et al. 2017). Multi-task learning approach has been used for a variety of research areas, i.e., sentence classification and tagging (Wang et al. 2020), entity recognition and semantic labeling (Alonso and Plank 2017), and two different financial forecasting (Yang et al. 2020). None of these studies used multi-tasking in POI recommendations. Halder et al. (Halder et al. 2021) proposed a transformer-based multi-task learning model for the next top-k POI recommendation and predicted queue time. This model can not capture user interests appropriately and can not solve the new POI cold start problem.

### Feature embedding

Feature embedding is another important factor in POI recommendation. The objective of feature embedding in the POI recommendation is two-fold: POI features embedding and user feature embedding. The main goal of POI feature embedding is to learn an encoding for POI network that effectively captures a POI’s crucial properties (i.e., their neighborhood POI distance, recent check-in, etc.). Similarly, user feature embedding is to learn an encoding that can capture user visiting behavior property. Most of existing studies  (Huang et al. 2019; Zhou et al. 2019a; Halder et al. 2021) used unique user identity as a user feature and could not appropriately capture user behavior. In this paper, we measure user similarity based on their previous visiting POI description similarity. This encoding is projected and processed into a low-dimensional space. Context-aware hierarchical POI embedding model using textual, visual, user, and temporal features have been proposed in (Chang et al. 2018a). Zhou et al. (Zhou et al. 2019b) introduced a more generic framework for POI recommendation that is sufficiently flexible to incorporate different contextual information. However, these models do not consider spatial influences. Several recent studies  (Feng et al. 2015; Hang et al. 2018; Yin et al. 2017) have shown how to embed items into a low dimensions space based on feature inner product. Chen et al. (Chen et al. 2020) showed that POI description-based similarity instead of only category-based similarity performs well and can handle new POI cold start problem efficiently. We are more interested in personalised next POI recommendation. Therefore, inspired by their research (Chen et al. 2020), we construct user interests-based POI visit sequence similarity using POI description.

### Differences from previous studies

Our proposed next POI recommendation with queuing time and user interest model differs from state-of-the-art POI recommendations in various aspects. First, we introduce complex spatiotemporal dependencies along with POI sequence in transformer model. The transformer model can capture nearby and long-distance POIs visiting relationships efficiently. Second, we present multi-task learning in POI recommendation that can recommend top-k POI and predict queuing time simultaneously. The queuing time may change user intentions. Third, to handle new category POI cold start problem, we measure POIs similarity based on POI description instead of category. The following Table 1 depicts the fundamental differences between our proposed model variants and baselines in terms of various constraints.

**Table 1:** Comparison between proposed models and various baselines, in terms of considering various constraints

	Models	Spatio-temporal	User Interest	Queue Time	Technique	Multi-tasking
Baselines	ST-RNN (Liu et al. 2016)	✓			LSTM	
	STACP (Rahmani et al. 2020)	✓			Matrix Factorization	
	APOIR (Zhou et al. 2019a)	✓			Adversarial	
	ATST-LSTM (Huang et al. 2019)	✓			Attention + LSTM	
	TLR (Halder et al. 2021)	✓			Transformer	
	TLR-M (Halder et al. 2021)	✓		✓	Transformer	✓
Proposed	TLR_UI	✓	✓		Transformer	
	TLR-M_UI	✓	✓	✓	Transformer	✓

## Preliminaries and problem statement

In this section, we first describe key preliminary definitions and then describe the problem statement.

### Definition 1

Point of Interest (POI): A POI *p* is defined as a uniquely identified location (e.g., roller coaster, museum, hotel and etc.) that can be geolocated based on its longitude and latitude. A sequence represents a set of POIs visited by a user, *P* = $\{p_1$, $p_2$, $\cdots$, $p_n\}$ that user visits sequentially.

### Definition 2

Visit Activity: User visit activity is a quadruple $$v^u_{t_k}$$ = $$(p^u_{t_k}$$, $$l^u_{t_k}$$, $$t_k, u)$$ which represents a user *u* visiting POI $$p^u_{t_k}$$ at location $$l^u_{t_k}$$ at timestamp $$t_k$$.

### Definition 3

Visit Sequence: A user visit sequence is a set of visit activities of the user, represented by $V_u$ = $\{ v^u_{t_1}$, $v^u_{t_2}$, $\cdots$, $v^u_{t_i}\}$. All user historical visit sequences in a dataset are defined by $V^U$ = $\{ V_{u_1}$, $V_{u_2}$, $\cdots$, $V_{u_{|U|}}\}$, here |*U*| is the number of all users.

### Definition 4

Visit Trajectory: A user visit trajectory is a subset of user visit sequence i.e. $V_{u}$ = $\cup _i S^u_i$, represented by $S^u_i$ = $\{v^u_{t_k}$, $v^u_{t_{k+1}}$, $\cdots$, $v^u_{t_{k+n-1}}\}$, where sequence length is n. In the sequence, if the time difference between two consecutive POI visits is more than six hours, we divided it into different trajectories. We ignored all the isolated POI (only one POI belongs in a sequence).

### Definition 5

Queuing Time Trajectory: The queuing time is a triplet $$q^p_{T_k}$$ = $$(p^u_{T_k}$$, $${T_k}$$, $$q_i)$$ representing that user *u* needs to wait $$q_i$$ time to access the POI $$p^u_{T_k}$$ at timestamps $$T_k$$. The queuing time sequence is a set of queuing time triplet $$S_q^{u_i}$$ = $$\{q^p_{T_k}$$, $$q^p_{T_{k+1}}$$, $$\cdots $$, $$q^p_{T_{k+n-1}}\}$$. All queuing time trajectories are indicated by $$Q^U$$ = $$\cup _i S_q^{u_i}$$, where $$u_i \in U$$. The length of visit sequence and queuing time sequence will be same. The timestamps $$T_k$$ can be fixed time units, e.g., an hour or half hour-based time interval.

### Definition 6

User Interests: Let user *u* visit a set of POIs, e.g., $\left\{ p_1, p_2, \cdots , p_n \right\}$. The POI $p_i \in P$ belongs to a specific relationship among other POIs based on POIs description. POIs descriptions effectively measure the relationship between two different categorical POIs and various interest levels among the same categorical POIs. Therefore, the user interests from $p_i$ to $p_j$ is defined as:

1 $$\begin{aligned} UI_{p_i,p_j} = Similarity(Description_{p_i}, Description_{p_j}) \end{aligned}$$

where, *Similarity*(.) measures the similarity between two POIs based on their descriptions. The intuition of this definition is that the visitor’s interest might be strongly connected with new categorical POIs in the system network.

**Problem Statement:** Given the input of all user visit trajectories $V^U$, queuing time trajectories $Q^U$ during past *T* timestamp and POI description, the output of our proposed multi-task learning model is to recommend next top-k POIs to the users and predict the prospective queuing time of recommended POIs, simultaneously. The model can recommend a fixed set of POIs (top-k) and can optimize queuing time between original time and predicted queuing time.

## Proposed models

In this section, we describe two proposed model variants that are user interest aware transformer-based multi-task learning recommendation **(TLR-M_UI)** model and user interest aware transformer-based learning recommendation **(TLR_UI)** model. We describe each component of our proposed models in the following sections.

### The TLR-M_UI model

**Fig. 2:**
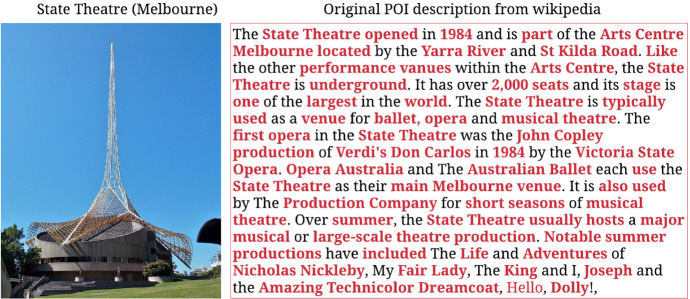
POI description

**Fig. 3:**
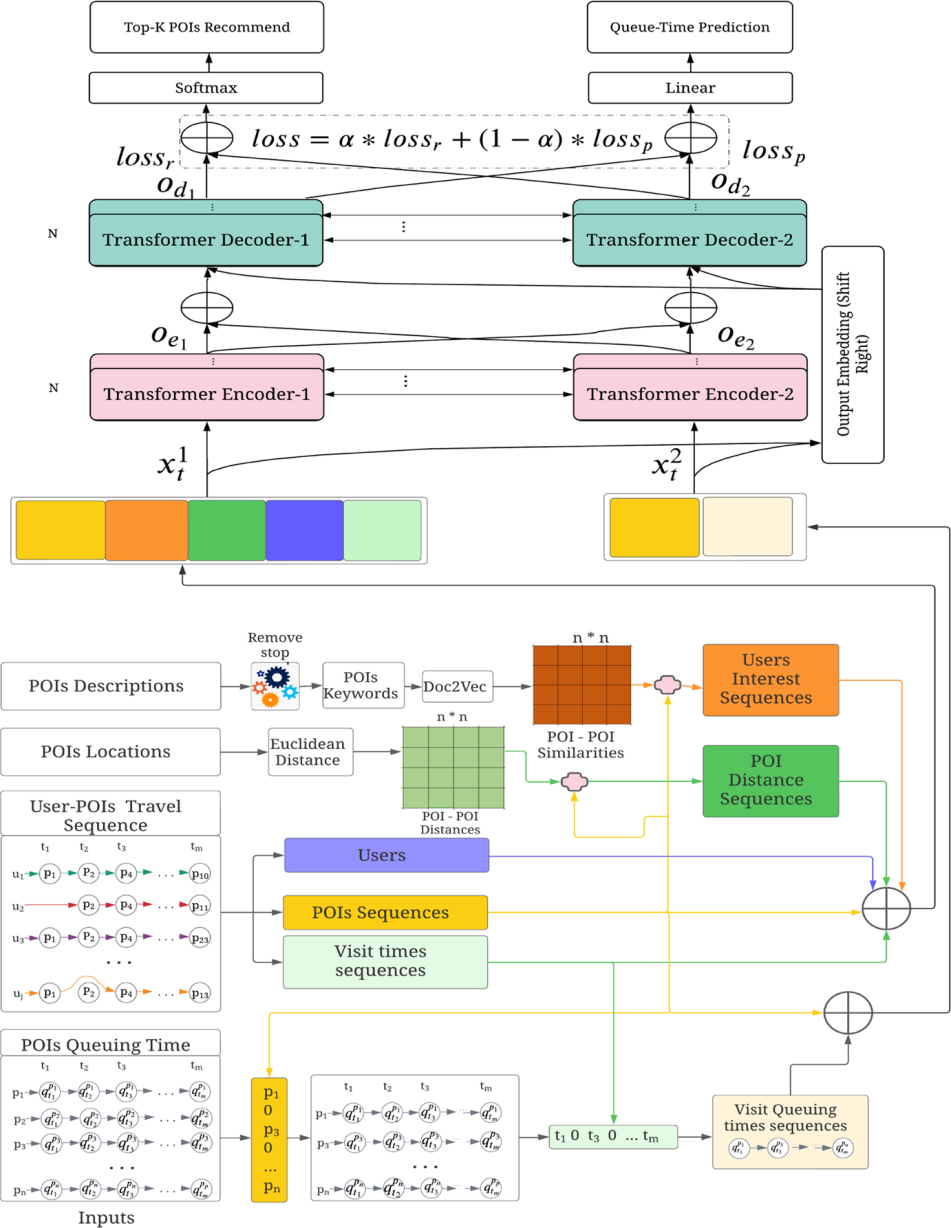
The architecture of proposed TLR-M_UI model

This section describes our proposed user interest aware transformer-based multi-task learning recommendation **TLR-M_UI** model. The model mines POI description-based user personalised interest and uses multi-head attention-based transformer. Figure 3 illustrates the architecture of TLR-M_UI model. The model takes POIs descriptions, POIs locations, POIs sequence and POIs queuing time information. The proposed model captures user interests based on POI to POI description similarities. User interest plays a significant role in personalised POI recommendations. Previous studies (Lim et al. 2017; Halder et al. 2022) utilised POI categorical interests that are unable to accurately capture POIs similarity when new categorical POI is added into the network and faces a new POI cold start problem. To solve the new POI cold start problem, Chen et al. (Chen et al. 2020) utilise textual information. Inspired by the performance of textual information, we measure user visiting POI to POI description similarity sequence and incorporated it in our previous proposed TLR and TLR-M models (Halder et al. 2021) to improve personalised POI recommendation. Figure 2 depicts the textual information of the State Theatre of Melbourne. We crawled POIs descriptions as textual information using Wikipedia API in this work. The textual information consists of POI features, categories, history, layout, etc., which are highlighted in bold red where standard English stop words are ignored.

From this example, we can observe that POIs from different categories may still share some similarities in various aspects despite being of a different POI category. We use Doc2vec (Le and Mikolov 2014) model to evaluate the similarity between POIs descriptions. After getting textual information of each POIs, we measure POIs description-based similarity to measure the POI-POI similarity.

Figure 3 shows that we get user interest sequences based on user visited sequences patterns and POI-POI similarities matrix. The POI recommendation also depends on spatial distance (Huang et al. 2019; Rahmani et al. 2020). We construct distance sequences based on POI sequences and their distance matrix to capture the spatial dependencies. To calculate the distance matrix, we use Euclidean distance. It has been observed that visitors’ preferences may change based on time. That means if visitors start their tour in the morning, it would be different if they start their tour in the afternoon. Thus, temporal influence is significant for POI recommendations. We construct temporal visit sequences from POI visit sequences to capture temporal dependencies. This tour should be personalised; one user interests may differ from the other user. In the model, unique user identity is used as a personalised indicator. Besides these, visitors are concerned about POI queuing time. Most of the time, they want to avoid longer queuing. To train our model, we also use POI sequence-based temporal sequence to represent queuing time sequence from the POI queuing time information.

Our proposed model first embeds all inputs into latent vectors at this input stage, then TLR-M_UI model uses these latent vectors. The model can not take string directly. That is why we used *n* dimensional space embedding. In our proposed model, our goal is to perform multi-tasking operations that recommend top-k POIs and simultaneously predict queuing time. Top-k POI recommendation depends on user personalised interest and spatio-temporal dependencies. On the other hand, predicting queuing time depends on POI and their queuing time duration. Here, we want to get the best-personalised recommendation with less queuing time. Thus, we need to combine these two objectives in one model. Transformer-based learning approaches have been shown significant improvement in natural language processing (NLP) tasks and volatility prediction (Yang et al. 2020; Wu et al. 2020). Transformer present state does not rely on the past hidden state like RNN and LSTM. It allows parallel computation, which reduces training time. It also reduces the drop of long dependency performance due to using the whole sentence instead of word-by-word dependencies. Moreover, positional embeddings and multi-head attention capture information about the relationship between different words. Inspired by the advantage that multi-head attention-based transformer has demonstrated over CNN or RNN/LSTM based deep learning models, we designed our next POI recommendation model based on the multi-attention-based transformer model. Thus, we apply two sets of Encoder and Decoder to perform multi-tasking. For a personalised recommendation, we use five inputs that express user personalised interest with spatio-temporal dependencies in *Encoder-1* and POI sequence and corresponding queuing time feature tuples input to transformer *Encoder-2*. Concatenating these features using the multiplication of five transition metrics, we obtain $x^1_t$ as input as follows:

2 $$\begin{aligned} \begin{aligned} x^1_t ={}&W_p p^u_{t_i} + W_l l^u_{t_i} + W_{t} t^u_i + W_u u_{t_i} + W_{in} u_{in} \end{aligned} \end{aligned}$$

where $p^u_{t_i}$, $l^u_{t_i}$, $t^u_i$, $u_{t_i}$ and $u_{in}$ represent POI IDs, spatial, temporal context, user vector and user interest respectively. $W_p$, $W_l$, $W_{t}$, $W_u$ and $W_{in}$ are transition matrices. The inputs represent all features as a real number in *n* dimensional space that that is fed as input to the transformer encoder. The main reason for these transition matrices is that the transformer encoder is unable to take POIs and features string input directly.

For queuing time prediction, we use tuples inputs which express queuing time dependencies in *Encoder-2*. Concatenating these queuing features and POI features using multiplication of transition metrics, we obtain $x^2_t$ as input as follows:

3 $$\begin{aligned} x^2_t ={}&W_p p^u_{t_i} + W_q q_{t_i} \end{aligned}$$

where $q_{t_i}$ is the queuing time at time $t_i$, $W_q$ is a transition matrix.

**Fig. 4:**
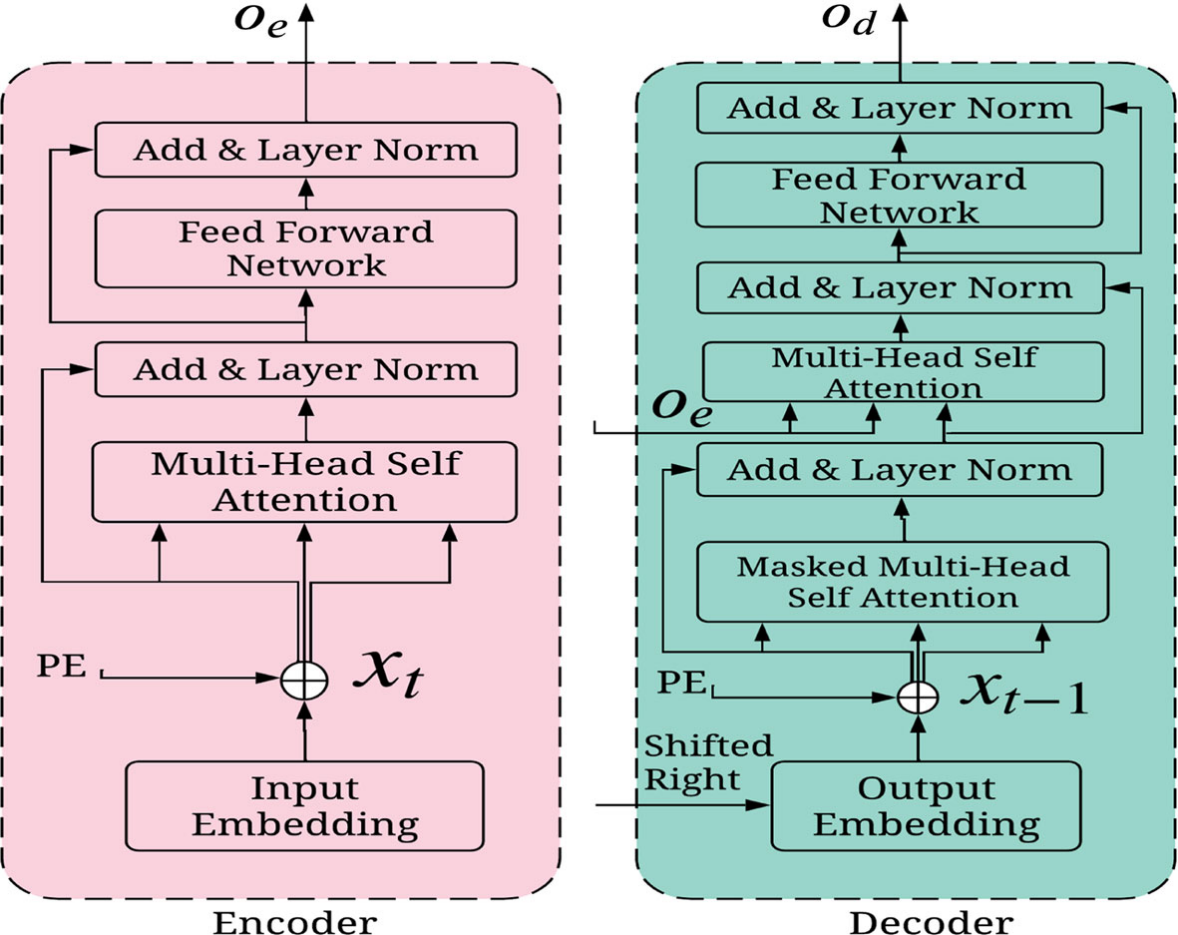
Standard encoder and decoder model

Each encoder consists of *N* layers, and each layer is composed of multi-head self-attention, fully connected feed-forward followed by layers normalization (Vaswani et al. 2017). Figure 4 shows the basic encoder architecture where first layer input comes from the element-wise addition between input embedding latent vector and positional encoding (Vaswani et al. 2017). Input embedding is represented by $x = (x_1, x_2, \cdots , x_m)$ with $x_t \in {\mathbb {R}}^f$ in which each $x_t$ is a column vector of the matrix embedding to the space ${\mathbb {R}}^{E_{size} \times f}$ where $E_{size}$ is embedding size and *f* is the features number of each embedding. The positional encoding plays a significant role to establish sequential data relationships without the use of RNNs or CNNs. The main idea is to add some consideration of sequential structure to the matrix embedding. To achieve that, we use positional embedding vector $PE = (PE_1, PE_2, \cdots , PE_m)$, where $PE_t \in {\mathbb {R}}^f$ with the input vector $x_t$. The resulting vector is $x = (x_1 + PE_1, x_2+PE_2, \cdots , x_m + PE_M)$. Each element of positional embedding vector value is calculated by the timing signal manner in (Vaswani et al. 2017) as follows:

4 $$\begin{aligned} \begin{aligned} PE_{pos, 2i} = \sin {\left( \frac{pos}{10000^{2i/E_{size}}}\right) } \\ PE_{pos, 2i+1} = \cos {\left( \frac{pos}{10000^{2i/E_{size}}}\right) } \end{aligned} \end{aligned}$$

where $E_{size}$ and *pos* denotes the embedding size and relative position of time value, respectively. We define 2*i* and $2i+1$ to indicate the embedding element index with the even and odd position, respectively. The output of first encoder layer is followed as input embedding in the next layer.

5 $$\begin{aligned} O_e = lNorm(x+FFN(lNorm (x + MultiHead(Q,K,V)))) \end{aligned}$$

where *lNorm(.)* function represents layer normalization, *FFN(.)* means fully connected feed-forward network,* MultiHead(.)* describes multi-head attention mechanism and *x* is input containing input features and positional encoding feature. The layer normalization function (Ba et al. 2016) is batch size-independent normalization that computes the mean and variance of all summed inputs to the layer neurons. The *FFN(.)* is two linear transformations with *ReLU* activation that takes input from the layer normalization followed by multi-head attention mechanism. Position wise input latent vector is applied in this FFN module and the same process is repeated for as many POIs in the sequence. The *ReLU* activation function is used because of its less computational operation and ability to solve the vanishing gradient problem.

The attention-based neural network can capture the correlation between latent POIs feature and user feature representation without sequential propagation. In the proposed model, the spatial, temporal, and user inter-dependencies among the time and geographical locations are jointly considered using attentive learning. The main aim is attending relevant pairwise POIs distance, check-in, or visiting time steps in sequence visits. Therefore, the quantitative relevance of visiting different POIs by the user is captured automatically using a multi-head attention mechanism (Vaswani et al. 2017). The multi-head attention mechanism aggregates the H times learning process indexed from 1 to h. Each head $h_i$ is calculated by the attention model, which can be formally represented as follows.

6 $$\begin{aligned} h_i = Attention (q,k,v) = softmax\left( \frac{q k^T}{\sqrt{d}}\right) v \end{aligned}$$

where *q* represents the one position input latent representation, *k* represents all the input vector representation and *v* indicates the full vector representation. Here, *d* is the dimension of a query as well as the key and value. The attention changes *v* value in encoder and decoder that are defined by each POIs are influenced by all the other POIs in the sequence considering vector representation. Additionally, the softmax function is used to define the influence value between 0 and 1. This attention can be parallelized *h* times with the linear projection of *q*, *k* and *v*. Thus, the system can learn different representation of *q*, *k* and *v* and automatically select the most beneficial one for the POI recommendation during the training time. Concatenating all heads, we find the output of multi-head attention as follows:

7 $$\begin{aligned} MultiHead(Q, K, V ) = Concat(h_1, h_2, \cdots , h_h) W_o \end{aligned}$$

where *Q*, *K*, and *V* are query, key, and value matrices that share the same input matrix and $W_o$ is the transition matrix. Multi-head query, key and values dimension will be *h* times larger than the attention model and equivalent to $E_{size}$. After the multi-head attention, we use a pointwise feed-forward layer that can describe the linear transformation of each POI from the given POI sequence. The feed-forward layer’s output in the encoder is fed as input to another layer and repeats *N* time. Then, the final representation of sequence learning is sent to the decoder as Q and K input.

Figure 4 shows the decoder which consists of six layers. Here, masked multi-head attention is used to avoid the current state from being generated again in the future. This characteristic is particularly useful for POI recommendation, where users usually do not like to visit the same place multiple times in a travel path or itinerary. Multi-head attention, feed-forward, and normalization layers are similar to the encoder. The decoder takes the same input as encoder input, with the information being shifted one position right to ensure that the prediction output of position $t_{i+1}$ only depends on available outputs up to time $t_i$. This output embedding is transformed by mask multi-head attention, multi-head attention, and feed-forward sub-layers using add and normalization functions. Each decoder’s output is repeatedly used as input in the decoder and transformed until *N* repetitions. The output is defined as follows:

8 $$\begin{aligned} \begin{aligned} O_d =&\, lNorm(x_{t-1}+FFN(lNorm (x_{t-1} \\&+\, MultiHead(O_e,O_e, MultiHeadMask(Q,K,V)))) \end{aligned} \end{aligned}$$

In this proposed model, we use two encoders and two decoders. The outputs of these two encoders are concatenated and fed into decoders that share the effect of top-k and queuing time together. *Decoder-1* and *Decoder-2* perform based on Equation 8. These two decoder outputs are updated using two different loss functions during the training phase. We used a soft parameter sharing architecture in which each task has its parameter setting-based model. These two model parameters are then regularized to reduce the difference among them and encourage them to be similar. We apply the multi-layer perceptron dropout technique to prevent over-fitting in the training phase. The dropout technique reduces the inner dependent learning among the neurons and offers a remarkable improvement of generalization error in network architecture (Srivastava et al. 2014). In the recommendation task, we use the softmax function to select the set of recommended POIs. Thus, the objective function for accurate top-k recommendation is as follows:

9 $$\begin{aligned} loss_r = - \frac{1}{N} \sum \nolimits _{i=1}^N [y_i log ({\hat{y}}_i) + (1-y_i) log ( 1 - {\hat{y}}_i)] \end{aligned}$$

where $y_i$ is the original output of TLR-M_UI model and ${\hat{y}}_i$ is the predicted output.

In the queuing time prediction component, we use Rectified Linear Unit *ReLU* as the activation function for the fully connected layer. We now compute the queuing probability corresponding to POI distributions. To reduce the difference between predicted probability and likelihood probability, we use the mean square error loss function as follows:

10 $$\begin{aligned} loss_q = - \sum \nolimits _{i=1}^N [(y^q_i - {\hat{y}}^q_i)^2] \end{aligned}$$

where $y^q_i$ and ${\hat{y}}^q_i$ represent original queuing time and predicted queuing time respectively.

Therefore, our objective function is a weighted average of these two loss functions using weight parameter $\alpha \in [0,1]$.

11 $$\begin{aligned} loss = \alpha \times loss_r + (1-\alpha ) \times loss_q \end{aligned}$$

We used Adam-optimizer (Kingma and Ba 2014) and applied the technique of decay learning rate with the steps until it reaches convergence. Adam optimizer combines two other extensions of stochastic gradient descent: Adaptive Gradient Algorithm (AdaGrad) and Root Mean Square Propagation (RMSProp) benefits. AdaGrad conserves a pre-parameter learning rate that improves sparse gradients’ problems performances. On the other hand, RMSProp preserves pre-parameter learning rates, which are maintained based on the average of recent gradients magnitudes for the weight. Decay learning rate trains the model with a significant learning rate and slowly reduces the rate until local minima. The decay helps both optimization and generalization. Finally, the TLR-M_UI model simultaneously performs our two desired tasks to recommend top-k POIs and predict their respective queuing time.

#### TLR-M_UI algorithm

Algorithm 1 presents TLR-M_UI model, which takes two sets of inputs, including POI sequence, spatiotemporal features, users, POI description based user interests and queue time feature. At the beginning of the Algorithm 1, we initialize all parameters in line 2. The algorithm defines POI to POI description-based similarity in line 3. POIs descriptions are paragraphs and sentence ordering might not be important. Thus, we use Doc2vec (Le and Mikolov 2014) to generate document/paragraph embeddings, which is used to compute POI description similarities based on the vectors. Then based on the batch size, we train our proposed model in lines 4-18. For each batch size, the algorithm takes two mini-batch inputs $x_b^1$ and $x_b^2$. After that, for each batch size, we find the user interests-based POI to POI sequence similarity in line 5. We combine the user interests with encoder inputs in line 6.
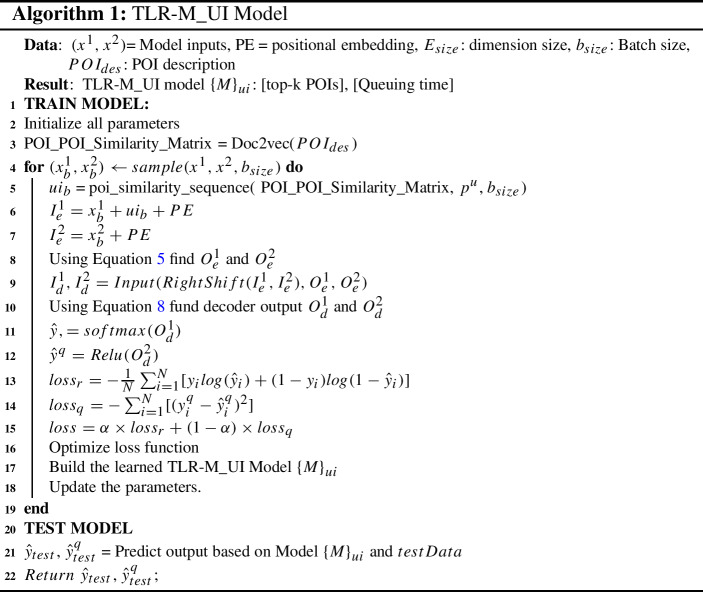


These inputs feed into the encoders and generate outputs using multi attention-based feed-forward network using Equation 5 in line 8. These two encoder outputs are fed as input into the decoders with right-shifted encoder inputs in line 9. After that, the multi-head attention layer’s output is normalized and added with the previous normalized layer output, passed to a fully connected feed-forward network, and subsequently two probabilities distributions are generated as outputs in line 10. The outputs are passed with softmax and rectified linear unit to compute the top-k POIs recommendation and queuing time prediction probabilities in lines 11 and 12. Using this probability, we apply two loss functions and achieve our objectives goal in lines 13 and 14. Furthermore, using the loss functions 13, 10 and 11 we train our proposed model $\left\{ M\right\} _{ui}$ and update all parameters in lines 17 and 18, respectively.

After constructing the model, we predict the next top-k potential POIs ${\hat{y}}_{test}$ using our test data and predict queuing time ${\hat{y}}^q_{test}$ in line 21. Finally, we measure our evaluation matrix based on output ${\hat{y}}_{test}$ and ${\hat{y}}^q_{test}$ compared to ground truth labels.

### The TLR_UI model

**Fig. 5:**
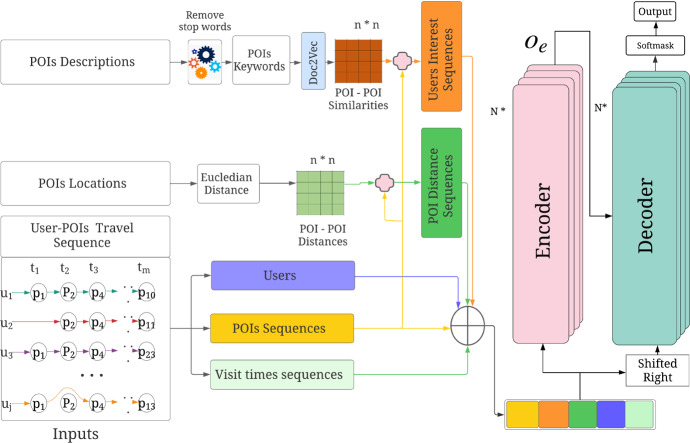
The architecture of proposed TLR_UI model

The TLR_UI model perform single task which takes input POI IDs, spatiotemporal and user latent features and user interest features. To model this information effectively, we use spatiotemporal information, user interests and POI sequence as input in the TLR_UI model to learn the non-linear dependency over the spatiotemporal context and POIs from historical tour activities. Figure 5 depicts the architecture of our proposed TLR_UI model.

The TLR_UI model takes five inputs as TLR-M_UI input. After that it passes into Encoder and Decoder. Finally, the output of the $N^{th}$ decoder is passed to the softmax layer to produce output probabilities of POIs. Among these output probabilities, top-k high-ranked POIs are recommended to the user. The output probabilities are defined as follows.

12 $$\begin{aligned} {\hat{y}} = softmax(O_d) \end{aligned}$$

Thus, the objective function for accurate top-k recommendation is to minimize the following equation:

13 $$\begin{aligned} loss_r = - \frac{1}{N} \sum \nolimits _{i=1}^N [y_i log ({\hat{y}}_i) + (1-y_i) log ( 1 - {\hat{y}}_i)] \end{aligned}$$

where $y_i$ is the original output and ${\hat{y}}_i$ is the predicted output. We used the Adam-optimizer (Kingma and Ba 2014) and applied the trick of decay learning rate with the steps until it reaches convergence.

#### TLR_UI algorithm

Algorithm 2 presents TLR_UI model, which takes inputs, including POI sequence, spatiotemporal features, users and POI description based user interests. At first, we initialize all parameters of the Algorithm 2 in line 2. Then, the algorithm defines POI to POI description-based similarity in line 3. We use Doc2vec (Le and Mikolov 2014) to generate document/paragraph embedding, which is used to compute POI description similarities based on the vectors. Then based on the batch size, we train our proposed model in lines 4-15. For each batch size, the algorithm takes mini-batch input $x_b^1$. After that, for each batch size, we find the user interests-based POI to POI sequence similarity in line 5. We combine the user interests with encoder input in line 6.
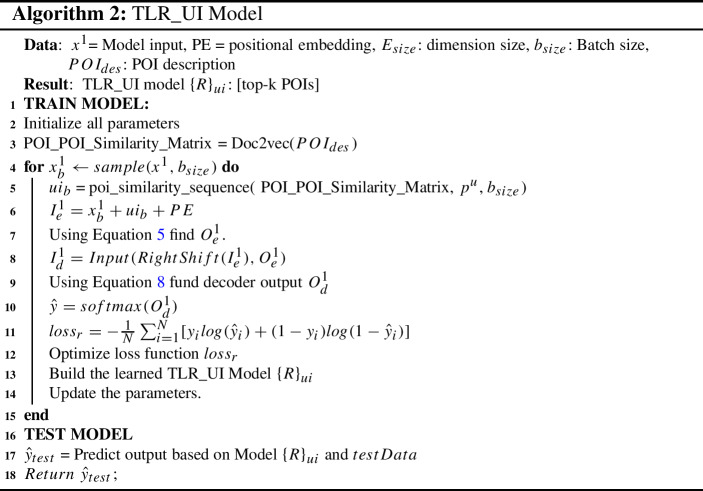


These inputs feed into the encoder and generate output using multi attention-based feed-forward network using Equation 5 in line 7. The encoder output is fed as input into the decoder with right-shifted encoder input in line 8. After that, the multi-head attention layer’s output is normalized and added with the previous normalized layer output, passed to a fully connected feed-forward network, and subsequently two probabilities distributions are generated as outputs in line 9. The outputs are passed with softmax to compute the top-k POIs recommendation in lines 10. Using this probability, we apply loss function and achieve our objective goal in line 11. Furthermore, using the loss function 13 we train our proposed model $\left\{ R\right\} _{ui}$ and update all parameters in lines 13 and 14, respectively.

After constructing the model, we predict the next top-k potential POIs ${\hat{y}}_{test}$ using our test data in line 17. Finally, we measure our evaluation matrix based on output ${\hat{y}}_{test}$ compared to ground truth labels.

### Computational complexity

All models, including baselines, predict the probability that a user will visit a POI based on the calculation of the dot products of user representation and POI representation. The models differ in user representation and POI representation at different time points. Thus, it is difficult to directly analysed the model time complexity based on the same parameters. We assume these models have the same dimension of hidden variable (represented by *m*), the same size of samples (represented by *n*), maximum number of check-ins *l*, length of time window (if required) *s*. Considering these variables we get ST-RNN time complexity is $O(nl (3sm^2+2m)+2nm )$ $\approx$ $O(nlm^2)$. Considering the attention weights, time complexity of ATST-LSTM model is $O(nl (9m^2+20m)+2nm )$ $\approx$ $O(nlm^2)$ (Huang et al. 2019). STACP model used Matrix Factorization to find the frequency matrix based on two low-rank matrices $U \in R^{m \times n}$ and $L \in R^{m \times l}$. Thus, the model complexity is *O*(*nml*). APOIR model used Matrix Factorization and Generative adversarial networks (GANs) for training data thus its total time complexity is *O*(*nml*). Our proposed models and existing baselines (TLR and TLR-M) used transformer architecture. Thus, this model complexity will be transformer architecture complexity. The transformer model’s complexity depends on multi-head self-attention and softmax function complexity. In the self-attention, each query assesses its compatibility with each key using a dot product. This dot product performs in a depth direction. Assume the query (Q) and key (K) dimensions are $n \times m$. The matrix multiplication of $QK^T$ is the product of matrix $n \times m$ multiplied by a matrix $m \times n$. Thus, the resulting complexity is $n^2m$. In softmax function, $n \times n$ matrix is multiplied by $n \times m$ matrix, which complexity is also $n^2m$. In the attention model, we need to focus on all POI which number is *l*, which is supposed to be much smaller than *n*. Thus, the transformer replace one self-attention on the whole input by n/l self-attentions on *l* places. Therefore, the total complexity of transformer model is $O(n \times l \times m)$.

In these four models, TLR, TLR-M, TLR_UI and TLR-M_UI complexities are same. Although different input feature numbers make the complexity variation. Therefore, these complexities are bounded by *O*(*nlm*). Table 2 shows the proposed models and baselines complexity analyses.

**Table 2:** Proposed models and baselines complexity analyses

Models	STRNN	ATST-LSTM	STACP	APOIR	TLR	TLR-M	TLR_UI	TLR-M_UI
Complexity	$O(nlsm^2)$	$O(nlm^2)$	*O*(*nml*)	*O*(*nml*)	*O*(*nlm*)	*O*(*nlm*)	*O*(*nlm*)	*O*(*nlm*)

## Experiments

In this section, we present experimental setup, datasets, baseline algorithms, and evaluation metrics. For these comparisons, our proposed (TLR-M_UI and TLR_UI) models and the existing baseline methods are implemented in the Python language. Training and testing sets selection are important factors in the deep learning model. At first, we construct an itinerary based on visiting POI where the first *t* steps are used as a model design and $t+1$ step is used as the next target POI. Thus, we construct all the prefixes of the input trajectories and make sub-trajectories as per standard practice (Tan et al. 2016). Difference in performance for our proposed models against baselines is evaluated for statistical significance using paired t-test. Experimental results show that TLR-M_UI significantly out-performs all baselines significantly ($p \le 0.035$).

### Environments

For these results comparisons, our proposed TLR_UI and TLR-M_UI models and the baseline methods are implemented in Python language. The experiments are run on 2.40 GHz Intel Core i5 with 8GB RAM in Windows 10. For the deep learning models, we have used TensorFlow and Keras libraries.

### Datasets

Our experiments were performed using six real datasets that are commonly used in tour recommendation research (Lim et al. 2017; Halder et al. 2021). The visit sequences of POIs are constructed based on photos taken time or check-in times to these POIs. If the time gap between two consecutive photos taken time or check-in time is greater than 6 hours, it is considered as a new visit sequence. For all other datasets, we filter out those users and POIs with fewer than 3 time visits and 3 visitors, respectively. For POI description, we collect the Wikipedia summary information using Wikipedia API based on POI name or POI longitude and latitude as search key. The variations of six datasets are shown in Table 3. In our experiments, the average performance of 10 runs is reported, wherein each run, we randomly select the training set using 70% random itineraries and the testing set using the remaining 30% itineraries. We know 10-fold cross-validation is better evidence of the performance analyses of the models. Therefore, here is a reason to select 70% training data and 30% testing data instead of 10-fold cross-validation. Our proposed model works based on personalised interest. In these datasets, many users have less than ten trajectories. If they all have at least ten trajectories, we could split training and testing data using 10-cross validation. Using 10-cross validation, we may train the model using one set of user interests data and test the model by applying another set of user interests. In our random selection, if users have only three trajectories, two trajectories are used to train the model and another one is used for testing purposes.

**Table 3:** Parameters description of various datasets

Dataset	# Photos/ #Check-in	POI Visits	# Users	# POIs
Epcot	90,435	38,950	2,725	17
Magic Kingdom	133,221	73,994	3,342	27
California Adventure	193,069	57,177	2,593	25
Budapest	36,000	18,513	935	39
Edinburgh	82,060	33,944	1,454	29
Melbourne	17,087	5,871	911	242

### Baseline algorithms

In this section, several baseline algorithms are described to compare the performance of our proposed TLR-M_UI and TLR_UI models that play a significant role in the next POI recommendation. Among a large number of existing works (Chen et al. 2018; Li et al. 2015; Feng et al. 2015; Cheng et al. 2012; Rahmani et al. 2020; Liu et al. 2016; Zhou et al. 2019a; Huang et al. 2019; Halder et al. 2021), we have considered several recent works as baselines that outperform the other baselines. We have used these baseline codes from GitHub that the authors shared. Therefore, the baseline algorithms related to our proposed algorithms are as follows.

- **ST-RNN** (Liu et al. 2016):^2^ A RNN-based next POI recommendation model that incorporates both geographical and temporal information within the recurrent framework.
- **STACP** (Rahmani et al. 2020):^3^ A matrix factorization based spatiotemporal activity centers model that jointly considers both geographical and temporal information.
- **APOIR** (Zhou et al. 2019a):^4^ An adversarial POI recommendation model that suggests POIs based on the learned distribution by maximizing the probabilities based on a rewarding framework.
- **ATST-LSTM** (Huang et al. 2019):^5^ An attention-based spatiotemporal LSTM based next POI recommendation approach that used spatiotemporal contextual information in check-in sequence.
- **TLR** (Halder et al. 2021):^6^ TLR is a multi-attention based transformer learning model model, which captures visitor’s historical check-ins spatiotemporal dependencies for POI recommendation.
- **TLR-M** (Halder et al. 2021):^7^ TLR-M is a transformer-based multi-tasking model, which simultaneously recommends POIs and predicts queuing time.

### Performance evaluation

To evaluate the performance of our proposed TLR-M_UI and TLR_UI algorithms and existing baseline algorithms to recommend the next POI, we consider a list of top-k recommended POIs for user *u* based on descending order of the probabilities. To show the performance of our models against the various baselines, we use the following standard metrics that were used in (Yin et al. 2017; Liu et al. 2017).

- **Precision@k:** Assume that $P_{r}$ be next POIs in the actual visit sequence and $P_{k}$ be the top k POIs recommended. The precision represents the ratio of the next top-k POI that is present in the original next POIs. We can define the Precision@k as follows.
  14 $$\begin{aligned} Precision@k = \frac{|P_{r} \bigcap P_{k}|}{|P_{k}|} \end{aligned}$$
- **Recall@k:** We use the same actual and recommended POIs $P_r$ and $P_k$ respectively. The Recall@k represents the ratio of real next POI that is also present in the top-k recommended POI for the user *u* and is defined as follows.
  15 $$\begin{aligned} Recall@k = \frac{|P_{r} \bigcap P_{k}|}{|P_{r}|} \end{aligned}$$
- **F1-Score@k:** The harmonic mean of both precision and recall of a user *u*, defined as follows.
  16 $$\begin{aligned} F1-Score@k = \frac{2 \times Precision@k \times Recall@k}{Precision@k + Recall@k} \end{aligned}$$
- **Normalized Discounted Cumulative Gain(NDCG@k):** NDCG evaluates the performance of next POI recommendation based on its position in the result list, it is defined as follows:
  17 $$\begin{aligned} NDCG@k = \frac{1}{U} \mathop {\sum }\limits _{u\in U} \frac{DCG@k(u)}{IDCG@k(u)} \end{aligned}$$
  18 $$\begin{aligned} DCG@k(u) = \mathop {\sum }\limits _{i = 1}^{k}\frac{2^{Rel_u}-1}{log_2(Ind_u + 2)} \end{aligned}$$
  where $Rel_u$ is 1 if *hit*@*N* = 1, otherwise it is 0. $Ind_u$ is the index of hit position that value is 0 to N-1. IDCG@k(u) is the ideal DCG@k(u) that means the index value is 0 to k-1.
- **Root Mean Square Error (RMSE)**: To evaluate the prediction accuracy of predicted queuing time and original queuing time, we use the RMSE evaluation metric as follows.
  19 $$\begin{aligned} RMSE = \sqrt{ \frac{1}{N}\sum \nolimits _{i=1}^N (y^q_i - {\hat{y}}^q_i)^2} \end{aligned}$$
  where *N* is the total number of queuing time prediction. $y^q_i$ and ${\hat{y}}^q_i$ represent actual and predicted queuing time respectively.

### Results and discussion

The performance of our proposed model TLR-M_UI and state-of-the-art POI recommendations are evaluated based on six dataset results. In all of these result analyses, we conduct experiments based on $\alpha = 0.5$.

#### Performance analyses

Table 4 shows the results of proposed models against the various baselines for the datasets in different evaluation metrics. The table shows that TLR-M_UI model outperforms the recent existing baselines in terms of all evaluation metrics, such as precision@5, recall@5, F1-score@5, NDCG@5, precision@10, recall@10, F1-score@10 and NDCG@10 results based on six datasets. It has been shown that proposed TLR-M_UI model outperforms all the single task models and multi-tasking model TLR-M. Among the single task model, it shows that TLR performs better among the baseline in top-k POI recommendations. Our proposed TLR_UI model outperforms all single task model in all datasets. Personalised user interests based single task model does not perform well in existing multi-tasking model TLR-M due to the advantages of multi-task learning which is relevant with POI recommendation. Therefore, our user interests based multi head attention based multi-tasking model TLR-M_UI outperforms significantly than then the multi-tasking TLR-M model. Furthermore, we perform our proposed models (TLR_UI and TLR-M_UI) with state-of-the-art POI recommendations are evaluated based on three research questions. In the next sections, we will describe each experiment in detail.

#### POI description based user personalised performance evaluation with single task baseline methods

The main criteria in evaluating POI recommendations is how accurately the recommended POIs reflect visitors’ actual visit POIs. Table 4 shows the performance of TLR_UI with single-task baseline models (all models except TLR-M and TLR-M_UI). It is clear that the proposed model significantly outperforms all single-task baselines. It is observed that the TLR model is the better baseline among the state-of-art baselines. Based on precision@5, TLR_UI outperforms the existing TLR POIs recommendation model on the Melbourne dataset to a maximum of 32.09% (TLR_UI model result is 0.0321 and TLR model is 0.0243). The same improvements can be observed in terms of recall@5 and F1-score@5 metrics. We also find similar improvement patterns at top k = 10 evaluation metrics.

It proves that user POI description-based personalised interest improves POI recommendation quality. The analysis of this experiment answers the first question. It shows that the POI description-based user interests and the transformer-based next POI recommendation model outperform the existing baseline models. The results also show that the values of evaluation metrics differ from dataset to dataset because we consider the top 5 and top 10 POIs among all POIs. Thus, the Melbourne dataset results show a low score compared to the other datasets because of the many POIs. Our results show the same pattern when we change the k value, i.e., k = 3 or 15.

**Table 4:**
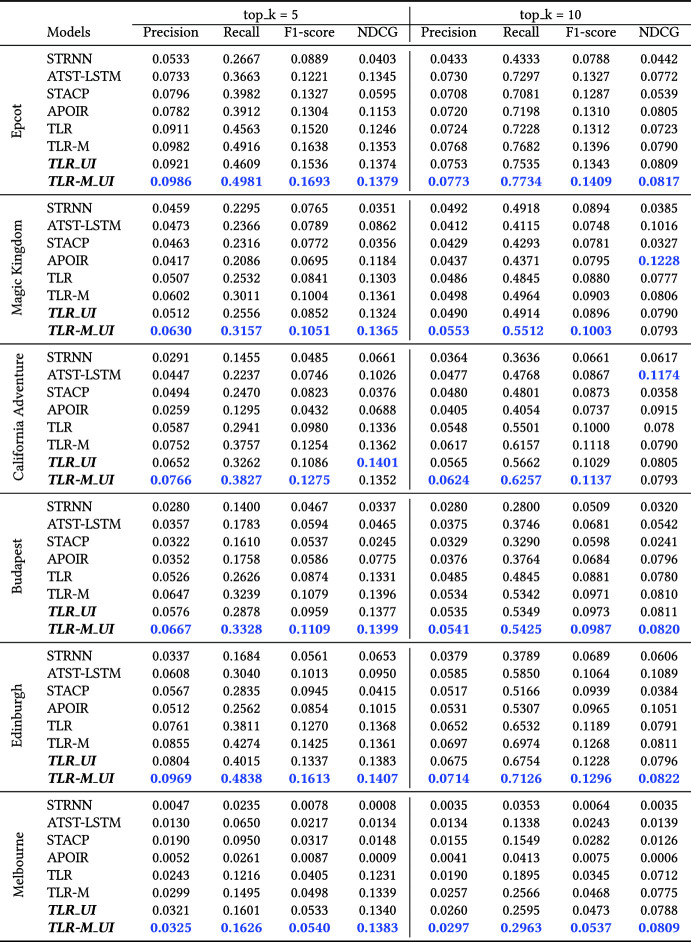
Results comparison among our proposed four models and various baselines, in terms of top_5 (Precision, Recall, F1-Score and NDCG) and top_10 (Precision, Recall, F1-Score and NDCG) evaluation metrics in six datasets. In each metric, higher values are better and the best result is highlighted by the bold numbers

#### POI description based user personalised performance evaluation with multi task baseline method

To the best of our knowledge, our previous paper (Halder et al. 2021) introduced queuing time prediction along with top-k POI recommendation simultaneously, which we term multi-task learning POI recommendation. Table 4 shows that the proposed user interests aware multi-task model TLR-M_UI outperforms all the baselines as well as our proposed user interests aware single task TLR_UI model. The user interest aware multi-task model TLR-M_UI outperforms on a maximum of 13.19% on the Edinburgh dataset and on average 5.68% in terms of F1-Score@5 compared to the TLR-M model. Similar kinds of improvements have been observed in F1-score@10 evaluation metrics. Earlier, we have seen that our proposed TLR_UI single task model outperforms all single-task baselines. The user interests aware multi-task learning model TLR-M_UI outperforms the single task TLR_UI model. We can see that TLR-M_UI outperforms on average 14.78% compared to the single task TLR_UI model in F1-score@5 and 7.98% in F1-score@10 metrics. All evaluations metrics show the same trends of improvement. Our proposed user interests aware multi-tasking model TLR-M_UI outperforms significantly compared to the existing baselines in other evaluation metrics. Therefore, these results show that user interests plays a significant role in improving the next POI recommendation. These analyses answer our second question, and we can say the user interests aware multi-tasking model performs well than the single task model and baselines.

#### Users interest performance analysis between POI description and category

In this paper, we introduce POI description-based user interests where existing works (Lim et al. 2017; Halder et al. 2022) applied POI categories based users similarities among the POIs. To evaluate the user interests based on POI categories, we developed TLR_UI(cate) and TLR-M_UI(cate) models where user interest sequences have been considered based on POIs categories. If a user moves a similar category of one POI to another, its value is 1, otherwise 0. Table 5 shows the TLR_UI outperforms TLR_UI(cate) and TLR-M_UI outperforms TLR-M_UI(cate) in terms of precision, recall and F1-score values. In the NDCG metric, except for the California dataset, our proposed TLR-M_UI outperforms best among the models. The main reason is that the POI description-based model can differentiate various interest levels within the same categorical POIs and find relationships among the POIs if they are not the same category. The categorical POIs face a cold start problem if a new category is introduced to the environment. Our proposed model can find similarities with existing POIs when new categorical POI is added to the environment. Thus, we said that our POI description-based user interest model outperforms the categorical user interest model.

**Table 5:**
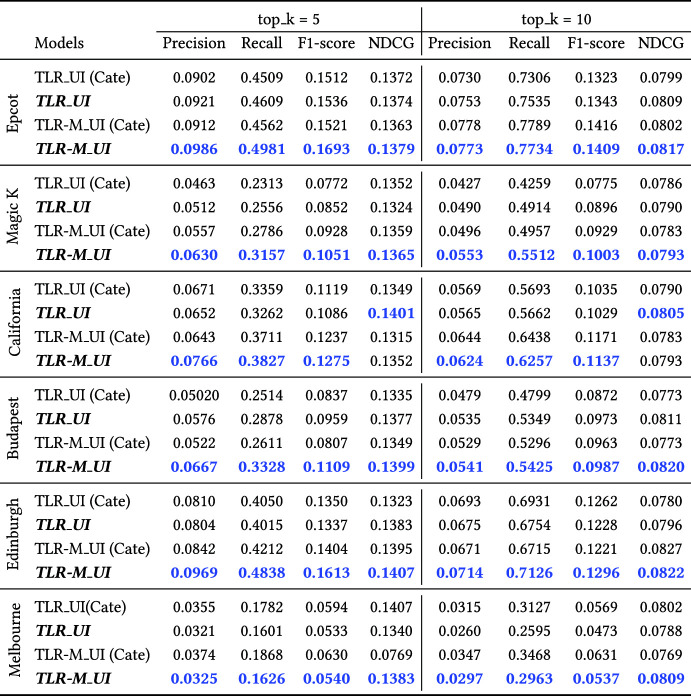
Results comparison between POI description vs. category based users awareness. In each metric, higher values are better and the best result is highlighted by the bold numbers

#### Performance of multi-task learning for queuing time prediction

The proposed TLR-M_UI model outperforms in terms of top-k POI recommendation and can also predict queuing time. The TLR-M_UI model underperforms the TLR-M model in queuing time prediction due to the consideration of user interest preferences. As these users prefer certain POIs that are more aligned to their interest preferences, they may have to wait a long time to reach their specific/preferred locations. To compare the TLR-M_UI model with a baseline prediction model, we use a single $TLR_q$ model that takes time-based queuing time and POI sequence as input and predicts target POIs queuing time as output. In this case, the RMSE loss function is used. We have developed an AT-$LSTM_q$ model applying the same input (queuing time and POI sequence) based on a variant of the ATST-LSTM (Huang et al. 2019) model. The ATST-LSTM$_q$ model cannot effectively predict the queuing time, as shown by RMSE value that is higher than the TLR-M_UI model values. Our proposed model’s queuing time prediction performance is higher than the TLR-M model because we consider user interests. We know users generally agree to wait a little longer to visit their preferred locations (Table 6).

**Table 6:** RMSE results between single and multi-task queuing time prediction. In each metric, lower values are better and the best result is highlighted by the bold numbers

Dataset	ATST-LSTM$_q$	TLR$_q$	TLR-M	TLR-M_UI
Epcot	1319.9	173.09	**102**. **48**	107.50
Magic Kingdom	925.6	90.48	**84**. **69**	89.05
California Adventure	1834.2	108.68	**101**. **50**	106.92
Budapest	2157.5	147.33	**129**. **17**	136.92
Edinburgh	1755.3	136.65	**113**. **19**	127.52
Melbourne	2602.5	132.44	**88**. **793**	127.35

#### Execution time comparison

Table 7 shows the execution time comparison for the proposed model and baselines. In the baseline model the non-deep learning model STACP is the fastest for training. It is well known that deep learning model takes longer time to train and depends on the number of parameters. Therefore, based on the best parameter setting in the baselines, we evaluate all models in the same environment. It is clear that our proposed model took less time than the other deep learning models. The main reason is that the transformer model avoids recurrent neural networks training; it works based on attention and positioning weighted sequence learning. It also shows that multi-tasking takes a longer time than single-task time. The reason behind that is multi-tasking used two sets of encoder and decoder. Finally, our proposed models’ testing time is comparable to the baselines.

**Table 7:** Execution time (Second) comparison for the proposed models and baselines

Category	Models	Epcot	Magic K	California	Budapest	Edinburgh	Melbourne
*Training*							
Non Deep	STACP	29.80	36.46	27.51	11.09	19.67	10.99
Deep	STRNN	232.89	536	301.22	100.07	120.78	78.07
	ATST-LSTM	103.06	418.91	175.65	17.04	24.44	35.78
	APOIR	251.88	439.19	264.49	78.96	101.67	183.75
	TLR	54.09	58.98	84.38	83.98	75.53	95.86
	TLR-M	170.79	453.71	727.91	492.97	667.48	207.45
	TLR_UI	49.40	59.63	69.50	66.51	54.72	55.48
	TLR-M_UI	184.61	491.53	574.42	399.07	556.04	189.42
*Testing*							
Non Deep	STACP	2.36	5.31	3.02	2.53	2.61	13.24
Deep	STRNN	1.09	2.42	1.77	0.289	0.613	0.395
	ATST-LSTM	1.13	3.11	1.52	0.29	0.39	0.47
	APOIR	8.61	7.83	8.22	8.09	7.29	12.47
	TLR	3.22	3.11	4.51	3.40	3.45	3.57
	TLR-M	6.62	10.40	11.18	9.92	12.03	7.06
	TLR_UI	3.16	3.17	3.25	2.97	2.87	2.85
	TLR-M_UI	6.99	11.41	10.99	9.84	12.82	6.57

## Conclusion

In this paper, we introduced the problem of user interests aware next POI recommendation and proposed models to solve this problem. We proposed POI description based user interests with multi-head transformer-based multi-task learning recommendation model TLR-M_UI that incorporates sequential, spatial, temporal and queuing time influences for recommending top-k POIs and predicting queuing time simultaneously. By leveraging the attention technique instead of a RNN architecture, the model can capture whole trajectory dependencies directly and efficiently. The model able to solve POI cold start problem. Experiment results based on six datasets show that our proposed models significantly outperform the various state-of-the-art models.

We have studied the queuing time aware top-k POI recommendation problem and solve the new POI cold start problem in this work. However, the models face challenges to solve new user cold start problems. In future research, we will consider user social relationships to solve new user cold start problem and construct a full itinerary considering the budget time that users get maximum entertainment.

## Footnotes

1 The terms "user" and "visitor" are used interchangeably.

2 https://github.com/yongqyu/STRNN.

3 https://github.com/rahmanidashti/STACP.

4 https://github.com/APOIR2018/APOIR.

5 https://github.com/drhuangliwei/An-Attention-based-Spatiotemporal-LSTM-Network-for-Next-POI-Recommendation.

6 https://github.com/sajalhalder/TLR.

7 https://github.com/sajalhalder/TLR-M.
